# The mitochondrial genomes of *Macrotermes* termites endemic to Ethiopia

**DOI:** 10.1080/23802359.2025.2500520

**Published:** 2025-05-04

**Authors:** P. Conrad Williams, Ahmed Ahmed, Bianka Franks, Daniel Debelo, Kamal M. Ibrahim

**Affiliations:** ^a^ School of Biological Sciences, Southern Illinois University Carbondale, Carbondale, IL, USA; ^b^ Department of Applied Biology, Adama Science and Technology University, Adama, Ethiopia

**Keywords:** Fungus growing termites, Ethiopia, termite taxonomy

## Abstract

The study of the fungus-growing termites genus *Macrotermes* in Africa remains notably limited, with the genus harboring hidden diversity that has eluded conventional taxonomy. Characterization of the mitochondrial genomes of these termite species represents an important biodiversity resource that could assist in resolving taxonomic ambiguities. The mitochondrial genomes of the two samples we sequenced, nominally *Macrotermes subhyalinus* (SE7) and *Macrotermes herus* (WE3), are 16,374 and 16,372 bp long respectively. We report congruent gene order for the two samples. Phylogenetic analysis using all protein coding genes in the mitochondrial genome recovered our WE3 mitochondrial genome as sister to a West African *M. subhyalinus* (JX144937) while our *M. subhyalinus* (SE7) mitochondrial genome is sister to *M. falciger* (KY224460). Our findings largely corroborate previous phylogenetic work on African *Macrotermes* and highlight the need for taxonomic scrutiny and increased taxonomic sampling of mitochondrial genomes within the clade.

## Introduction

Over 1000 species of termite have been identified in Africa alone, with 61 species specifically documented in Ethiopia (Cowie et al. 1990). The genus *Macrotermes* is well known for building large, architecturally intricate mounds in the region, but no representatives from that region have had their mitochondrial genomes sequenced. Of the 16 mitochondrial genomes for *Macrotermes* available on GenBank and identified to species, only 5 are from Africa. Further characterization of the mitochondrial genomes of these termite species, especially within the African clade, represents an important resource for resolving taxonomic ambiguities.

The ranges of the currently identified Ethiopian termite species span the whole country except for *Macrotermes herus* which is the sole species that is restricted to Western and Central Ethiopia (Debelo 2018). The range of *M. subhyalinus* is extends beyond Ethiopian borders. Within this vast range, substantial variations in mound structures among colonies have been observed. These disparities in mound architecture, combined with molecular data, suggest that the species as presently classified most likely comprises multiple non-sister species (Brandl et al. 2007; Egan et al. 2021). The complete mitochondrial genomes of two termites that were nominally identified as *Macrotermes subhyalinus* (Rambur, 1842) and *Macrotermes herus* (Sjöstedt, 1914) from Southern and Western Ethiopia, respectively, are reported here.

## Materials and methods

### Sampling

**Figure 1.**
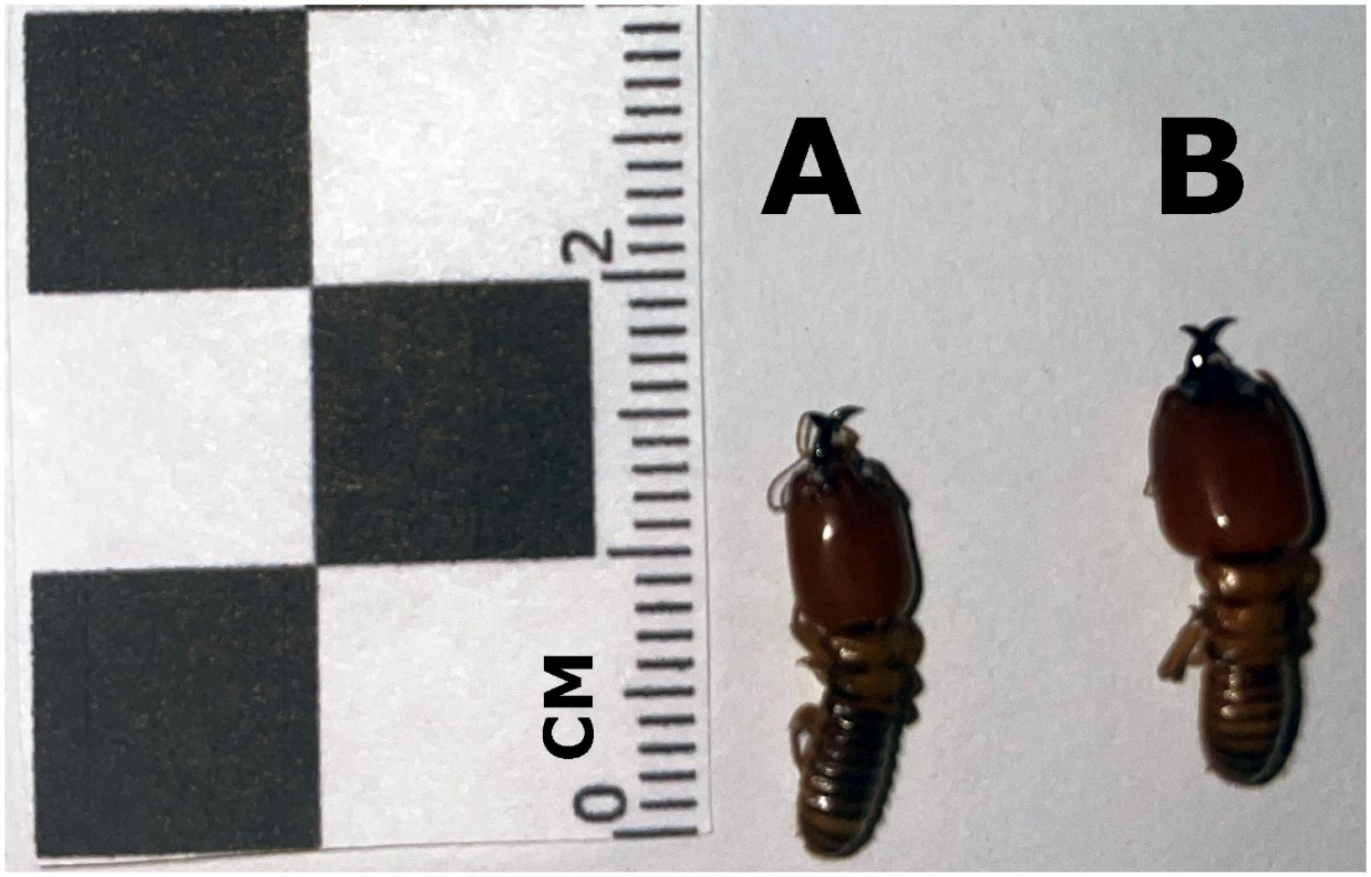
Dorsal view photo of *Macrotermes subhyalinus* specimen SE11 (left; A) and *Macrotermes herus* specimen WE1 (right; B). SE11 is the same species as SE7, *Macrotermes subhyalinus*, and WE1 is the same species as WE3, *Macrotermes herus*, as supported by COX1 barcode region sequencing (Ahmed 2021). Squares on the size reference are 1 cm^2^. Ventral view available in Supplement Figure S3. These photos were taken by P. Conrad Williams.

Termites were collected from mounds in western and southern Ethiopia as part of a survey of their taxonomic and ecological diversity in the region (Debelo 2018). Sample SE7 was collected at latitude 5.1235 and longitude 38.2863. Sample WE3 was collected at latitude 9.6855 and longitude 35.2705. The collected samples were identified at the genus level as *Macrotermes* based on their morphological characteristics and initially identified to species based on external mound morphology. Specifically, *M. herus* mounds were identified by their low to the ground and flat with closed tops while *M. subhyalinus* mounds were identified by being low to the ground with open turrets. Species identification was confirmed using COX1 barcode sequences obtained from Sanger sequencing, as detailed in Ahmed (2021). In brief, we constructed a phylogeny based on COX1 barcode sequences to confirm that our samples were sister to individuals of the same species that they were assessed to be based on mound morphology in the field (Supplement Figure S4). Two of these were selected for mitochondrial genome sequencing that we are reporting here. Sample WE3 from western Ethiopia was identified as *Macrotermes herus* and sample SE7 from southern Ethiopia was classified as belonging to one of several non-sister clades of *Macrotermes subhyalinus*, specifically the East African clade as described in Brandl et al. (2007). Example photos of both species from other individuals collected at the same time and identified to species using the COX1 barcode are shown in Figure 1. The two specimens were vouchered at the Illinois Natural History Survey Insect Collection (McElrath 2024; tcm@illinois.edu) under voucher numbers 1538544 (SE7; *Macrotermes subhyalinus*) and 1538545 (WE3; *Macrotermes herus)*.

### DNA extraction

DNA was extracted from two to three legs severed from each termite using the GeneJET Genomic DNA Purification Kit (Thermo Scientific), quality checked by electrophoresis in 1.5% agarose gel and quantified using a Qubit Fluorometer (Thermo Scientific) prior to sequencing. Library preparation, short read shotgun sequencing, and removal of adaptors was performed by the Roy J. Carver Biotechnology Center at the University of Illinois Urbana-Champaign. Libraries were prepared using the Kapa HyperPrep library construction kit from Kapa Biosystems (Roche) with an insert size of approximately 400 bp. Sequencing of 250 bp paired-end reads was performed on a NovaSeq 6000 (Illumina) yielding 22,922,840 paired reads for sample SE7 and 24,143,002 paired reads for sample WE3.

### Mitochondrial genome sequencing and annotation

Attempts at *de novo* assembly resulted in omission of a known repeated sequence in the control region. Specifically, the regions annotated repeat unit B2 and B3 in JX144937 (*Macrotermes subhyalinus*), both 553 bp, proved difficult to recover in de novo assembly. These two regions have 100% identity to each other in JX144937 and are present in other mitochondrial genomes at lower sequence identity (e.g. 94.4% identity in KM405637 after alignment with MAFFT). Therefore, we opted for mapping the reads to the mitochondrial genome of *M. subhyalinus* obtained from the Genbank (JX144937). A random subset consisting of 20% of the paired reads for the two samples, SE7 and WE3, was obtained using seqtk 1.3 (Li). These were then mapped to the reference mitochondrial genome using Bowtie v2.45 (Langmead and Salzberg 2012) as implemented in Geneious Prime 2023.1.2 (Kearse et al. 2012). We ran Bowtie 2 using its ‘–very-sensitive’ preset, excluding read pairs for which only one member of the pair successfully mapped. We then created a consensus sequence based on this mapping and mapped all reads to this consensus sequence using the same settings. Because BowTie2 is not aware that the mitochondrial genome is circular, coverage is artificially low near the ‘ends’ of the linearized reference sequence. To account for this, we created a second reference with the control region moved from the end of the linear reference sequence to the beginning and repeated the above-described mapping procedure. This allowed us to resolve base calls near the ‘ends’ of the reference mitochondrial genome. Coverage plots for both assemblies are shown in Supplement Figure S1 and S2. Finally, we used Geneious to annotate our mitochondrial genome based on similarity to *Macrotermes subhyalinus* reference. To compare our sample (SE7) from the East African *M. subhyalinus* clade to the reference mitochondrial genome from the West African *M. subhyalinus* clade, we calculated K2P distances using DnaSP (Rozas et al. 2017). Mitochondrial genome sequences were submitted to GenBank and are available under accession numbers OR653920 (SE7; *Macrotermes subhyalinus*) and OR653921 (WE3; *Macrotermes herus*).

**Figure 2.**
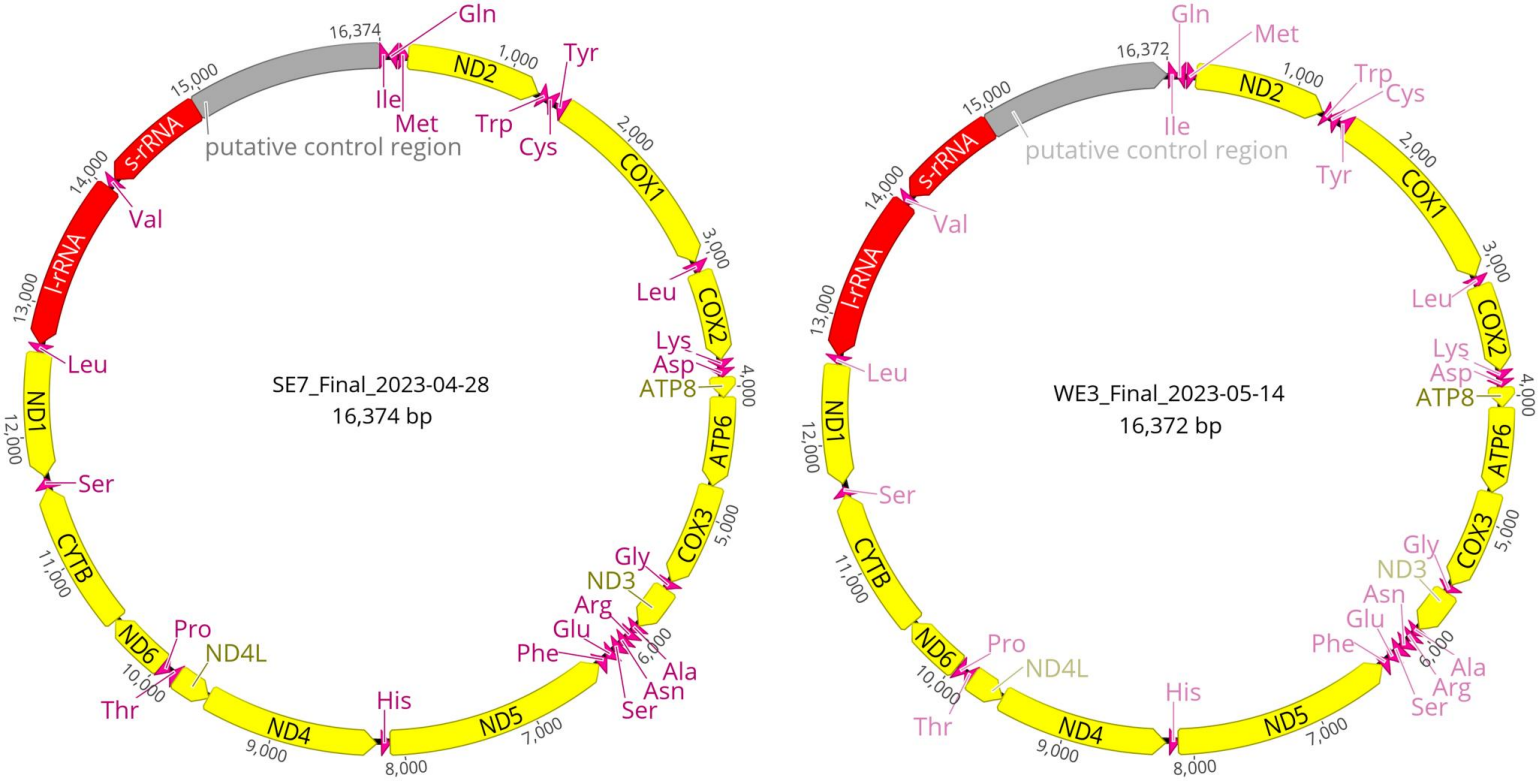
Annotated mitochondrial genomes for (top) sample SE7 (OR653920), *M. subhyalinus*, and (bottom) sample WE3 (OR653921), *M. herus.*

### Phylogenetic tree inference

To provide phylogenetic context for our newly sequenced genomes, we performed maximum likelihood tree inference. We downloaded all currently available *Macrotermes* mitochondrial genomes from GenBank. We aligned each of the 13 protein coding genes separately using MAFFT v7.526 (Katoh and Standley 2013). These alignments are provided in the supplemental materials. The best fitting substitution model for each protein coding gene alignment was determined using ModelFinder using the ‘-m TEST’ argument (Kalyaanamoorthy et al. 2017). Best-fitting nucleotide substitution models are reported in Supplementary Table S2. Finally, we used IQ-TREE 2 for maximum likelihood tree inference treating each protein coding gene as a separate partition and assessed support by performing 1000 ultrafast bootstrap replicates (Minh et al. 2020).

## Results

**Figure 3.**
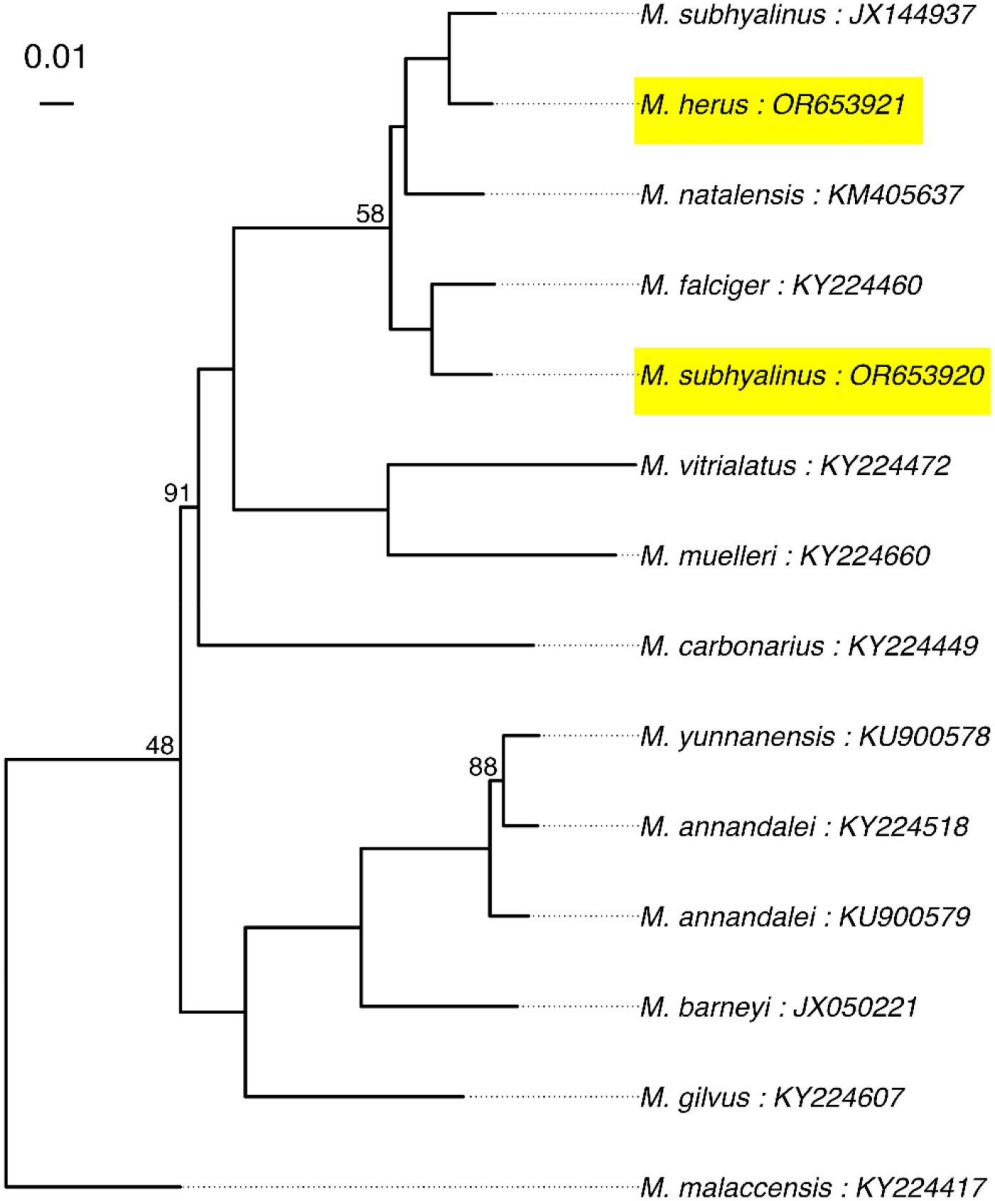
Phylogenetic tree showing the maximum likelihood phylogeny of *Macrotermes* based on the 13 protein coding regions of the mitochondrial genomes. The inferred tree was unrooted and the placement of the root along the branch to *M. malaccensis* is arbitrary. Our newly sequenced mitochondrial genomes are highlighted. Ultrafast bootstrap support values are shown on branchesBootstrap values equal to 100 are omitted for visual clarity. Sequences from Genbank are named using the species name and accession separated by colons (:). In addition to our newly generated sequences, the following GenBank sequences were used: *Macrotermes malaccensis* [KY224417] (Bourguignon et al. 2017), *Macrotermes barneyi* [JX050221] (Wei et al. 2012), *Macrotermes annandalei* [KU900579], *Macrotermes annandalei* [KY224518] (Bourguignon et al. 2017), *Macrotermes yunnanensis* [KU900578], *Macrotermes gilvus* [KY224607] (Bourguignon et al. 2017), *Macrotermes carbonarius* [KY224449] (Bourguignon et al. 2017), *Macrotermes muelleri* [KY224660] (Bourguignon et al. 2017), *Macrotermes vitrialatus* [KY224472] (Bourguignon et al. 2017), *Macrotermes falciger* [KY224460] (Bourguignon et al. 2017), *Macrotermes natalensis* [KM405637] (Meng et al. 2016), and *Macrotermes subhyalinus* [JX144937] (Cameron et al. 2012).

The *M. subhyalinus* mitochondrial genome (Sample SE7) is 16,374 bp long, and the *M. herus* mitochondrial genome (Sample WE3) is 16,372 bp long. Both genomes consist of 13 protein coding genes, 2 rRNAs, 22 tRNAs, and a putative control region (Figure 2). The phylogenetic relationship between our newly sequenced mitochondrial genomes and currently available *Macrotermes* mitochondrial genomes is shown in Figure 3. The average K2P distance between JX144937 and SE7 in the protein coding regions used to create the phylogenetic tree was 0.046 (Standard Deviation ±0.01; Supplement Table S1).

## Discussion and conclusion

Both mitochondrial genomes have the same gene order as all other mitochondrial genomes in the genus *Macrotermes* available *via* GenBank. The phylogenetic analysis using all mitochondrial protein coding genes recovered our M. herus (WE3) mitochondrial genome as sister to a *M. subhyalinus* mitochondrial genome (part of the western *M. subhyalinus* clade described in Brandl et al. 2007) while our *M. subhyalinus* (SE7) mitochondrial genome is sister to a M. falciger (Figure 3). Our phylogenetic analysis supports previous work reporting two non-sister clades of *M. subhyalinus* suggesting our current understanding of *Macrotermes* taxonomy needs re-evaluation. Our findings support the claim that what is currently described as *M. subhyalinus* may be multiple (2 or more) unique species, as suggested by Egan et al. 2021 and originally detected in Brandl et al. 2007. Further, our mitochondrial genome sequence for our *M. subhyalinus* specimen (SE7) is the first complete mitochondrial genome to be sequenced in the East African *M. subhyalinus* clade described by Brandl et al. 2007 (Egan et al. 2021 genetic group G1) while the *M. subhyalinus* mitochondrial genome from GenBank (JX144937) appears to come from the West African *M. subhyalinus* clade that is sister to *M. herus*. Our phylogenetic analysis was impeded by limited taxonomic sampling due to the limited number of African *Macrotermes* genomes available on GenBank. Efforts to build a more complete collection of *Macrotermes* mitochondrial genomes will aid in a variety of research efforts, primarily taxonomy, as well as increasing our understanding of the geographic range of both *M. subhyalinus and M. herus,* as well as that of the clade.

## Supplementary Material

Supplement_rev6.docx

## Data Availability

The genome sequence data that support the findings of this study are openly available in GenBank of NCBI at [https://www.ncbi.nlm.nih.gov]. The BioProject number associated with our samples is PRJNA1189520. For sample SE7, the newly generated mitochondrial genome is available on GenBank under accession number OR653920. The associated BioSample number is SAMN44979933, and associated reads are available in the Sequence Read Archive under SRR31452052. For WE3, the associated sequence accession, BioSample, and SRA numbers are OR653921, SAMN44979995, and SRR31452051, respectively. A list of other GenBank accession numbers used in the phylogenetic tree in this study is provided as a supplement. Voucher specimens are deposited at the INHS Insect Collection (McElrath 2024; tcm@illinois.edu) under voucher numbers 1538544 and 1538545.
