# Supplementary material for: The mitochondrial genomes of Macrotermes termites endemic to Ethiopia
Source: Mitochondrial DNA B Resour. 2025 May 4;10(6):420–4. doi: 10.1080/23802359.2025.2500520 (PMC12051524; doi:10.1080/23802359.2025.2500520)
Supplement: Supplement_rev6.docx [file TMDN_A_2500520_SM5873.docx]

# The mitochondrial genomes of *Macrotermes* termites endemic to Ethiopia: Supplementary Material

**P. Conrad Williams^1,3^, Ahmed Ahmed^1^, Bianka Franks^1^, Daniel Debelo^2^, Kamal M. Ibrahim^1^**

^1^School of Biological Sciences, Southern Illinois University Carbondale, Carbondale, IL 62901

^2^Department of Applied Biology, Adama Science and Technology University, P. O. Box 1888, Adama, Ethiopia.

^3^Corresponding author; [conrad.williams@siu.edu](mailto:conrad.williams@siu.edu)

Contents

[The mitochondrial genomes of *Macrotermes* termites endemic to Ethiopia: Supplementary Material 1](#_Toc190551438)

[Mitochondrial Genome Coverage Plots 2](#_Toc190551439)

[Ventral view of termite specimens 4](#_Toc190551440)

[K2P distances for SE7 vs. the M. subhyalinus reference (JX144937) 5](#_Toc190551441)

[Best fitting nucleotide substitution models 5](#_Toc190551442)

[Phylogeny based on COX1 barcode sequences 6](#_Toc190551443)

## Mitochondrial Genome Coverage Plots

**
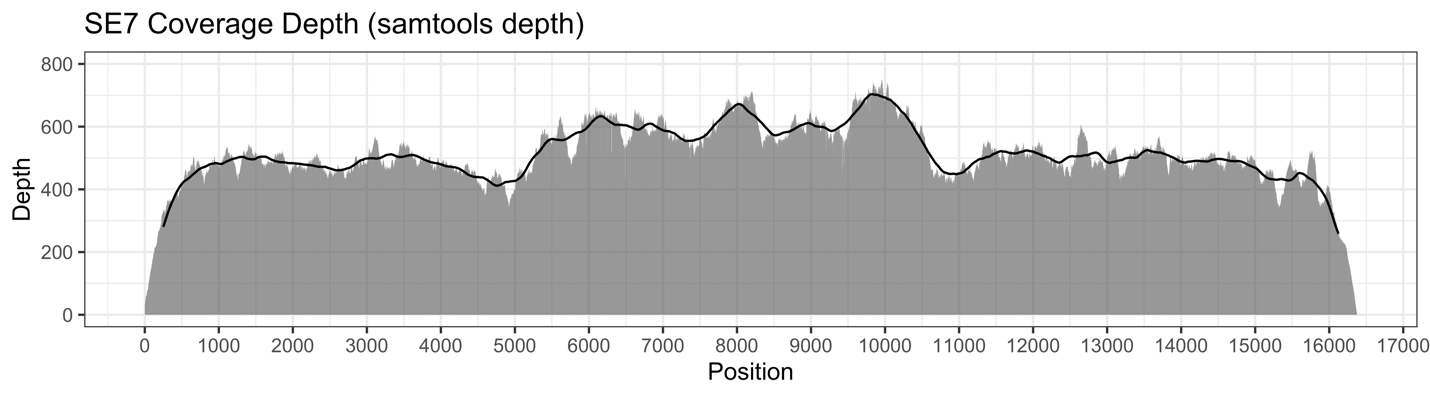
**


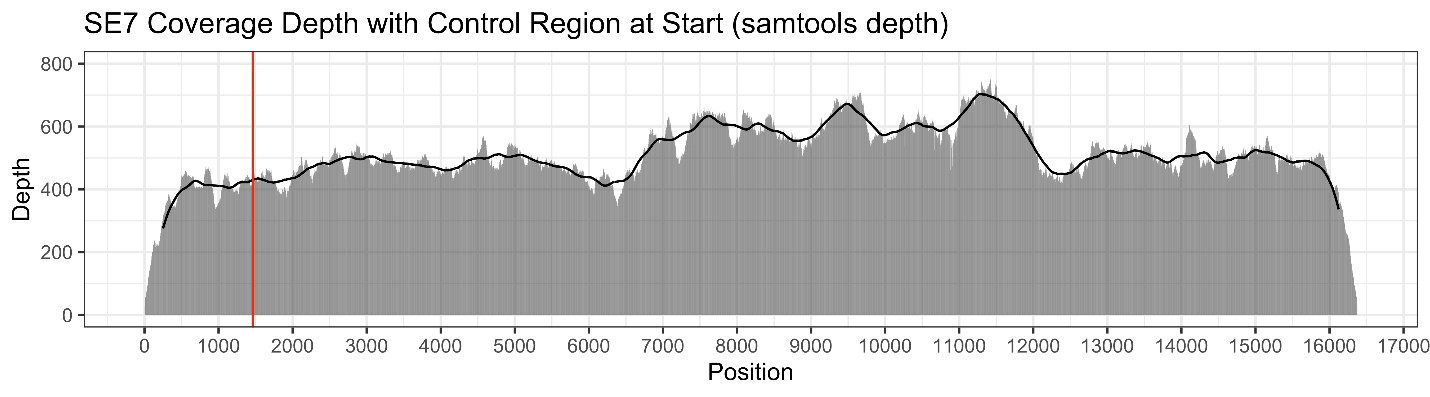


Figure S1: Depth of coverage plot for the SE7 mitochondrial genome. The grey histogram shows the read depth of the bowtie 2 mapping at each position of the mitochondrial genome as summarized by samtools depth. The black line represents the rolling average depth of coverage based on a 500 bp window. The top plot shows coverage based on bowtie2 mapping to the SE7 genome with the control region at the end of the sequence. The bottom plot shows coverage based on bowtie2 mapping to a different linearization of the SE7 mitochondrial genome created by moving the control region to the beginning of the sequence. The red line in the bottom plot shows the location of the end of the control region.

**
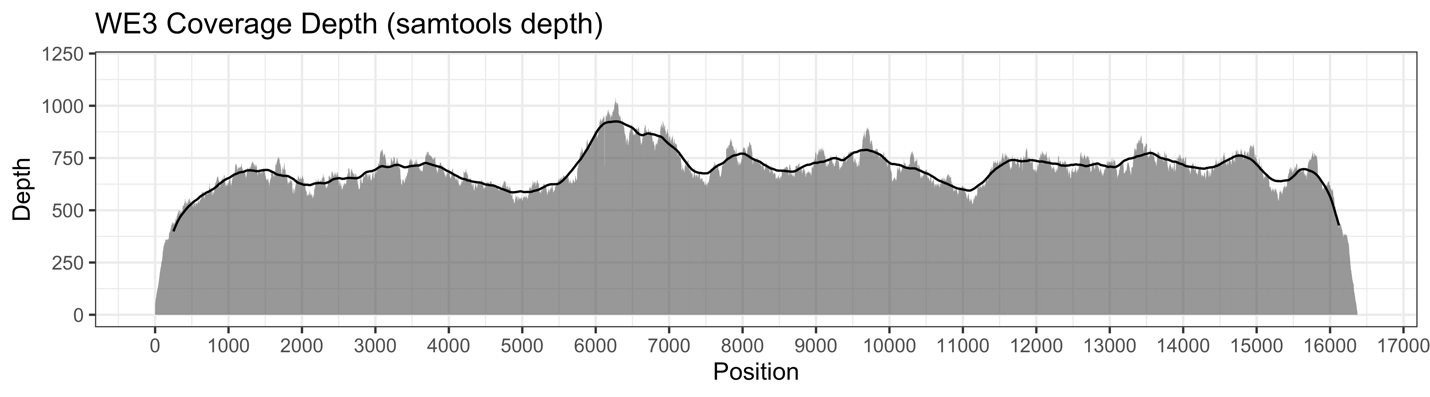
**


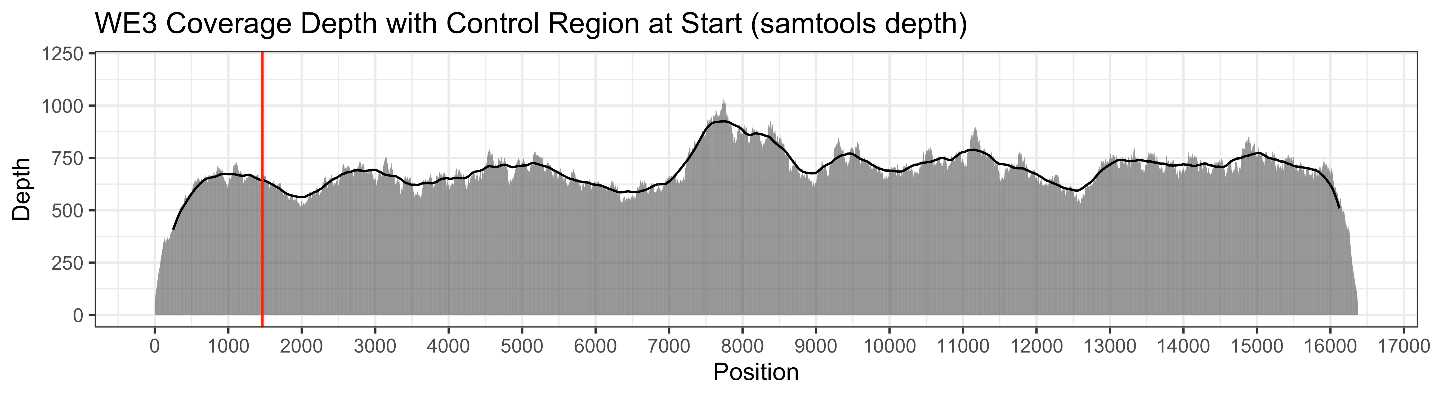


Figure S2: Depth of coverage plot for the WE3 mitochondrial genome. The grey histogram shows the read depth of the bowtie 2 mapping at each position of the mitochondrial genome as summarized by samtools depth. The black line represents the rolling average depth of coverage based on a 500 bp window. The top plot shows coverage based on bowtie2 mapping to the WE3 genome with the control region at the end of the sequence. The bottom plot shows coverage based on bowtie2 mapping to a different linearization of the WE3 mitochondrial genome created by moving the control region to the beginning of the sequence. The red line in the bottom plot shows the location of the end of the control region.

## Ventral view of termite specimens

**
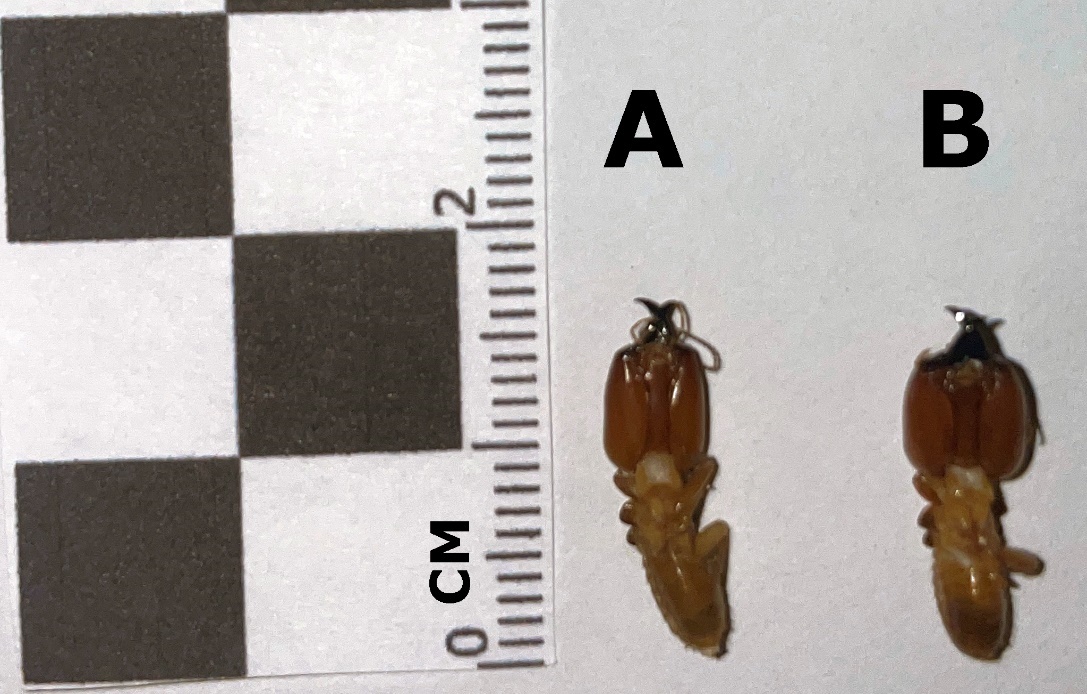
**

Figure S3: Ventral view photo of Macrotermes subhyalinus specimen SE11 (left; A) and Macrotermes herus specimen WE1 (right; B). SE11 is the same species as SE7, Macrotermes subhyalinus, and WE1 is the same species as WE3, Macrotermes herus, as supported by COX1 barcode region sequencing (Ahmed 2021). Squares on the size reference are 1 cm^2^ These photos were taken by P. Conrad Williams.

## K2P distances for SE7 vs. the M. subhyalinus reference (JX144937)

Table S1: K2P distances for each protein-coding region between SE7 (OR653920) and JX144937, both nominally M. subhyalinus but from the non-sister East African and West African clades, respectively.

| **Mitochondrial CDS** | **K2P Distance** |
| --- | --- |
| atp6 | 0.0502 |
| atp8 | 0.0529 |
| cox1 | 0.0487 |
| cox2 | 0.0614 |
| cox3 | 0.035 |
| cytb | 0.0512 |
| nad1 | 0.0374 |
| nad2 | 0.0408 |
| nad3 | 0.0348 |
| nad4 | 0.0336 |
| nad5 | 0.0463 |
| nad6 | 0.0617 |

## Best fitting nucleotide substitution models

Table S2: Best-fitting nucleotide substitution model for each mitochondrial CDS alignment as determined by ModelFinder using the ‘-m TEST’ option.

| **Mitochondrial CDS** | **Best Fitting Model** |
| --- | --- |
| atp6 | TIM2+F+G4 |
| atp8 | HKY+F+G4 |
| cox1 | TIM2+F+G4 |
| cox2 | TIM2+F+G4 |
| cox3 | TIM2+F+G4 |
| cytb | TIM+F+G4 |
| nad1 | TN+F+I+G4 |
| nad2 | K3Pu+F+G4 |
| nad3 | K3Pu+F+G4 |
| nad4 | TN+F+G4 |
| nad4l | TN+F+G4 |
| nad5 | TN+F+I+G4 |
| nad6; | K3Pu+F+G4 |

## Phylogeny based on COX1 barcode sequences

Below, we include a phylogeny based on COX1 barcode sequences to support our identification of the species and explain why our *M. subhyalinus* (SE7) is non-sister to the reference *M. subhyalinus* (JX144937; referred to by its RefSeq accession NC_018128 in the tree below) genome in the mitochondrial genome tree in the manuscript. While some nodes are only weakly supported, the taxonomic and geographic sampling of the COX1 barcode region is more complete than what is available using whole mitochondrial genomes and illustrates that *M. subhyalinus* as currently described is not monophyletic, as has been reported previously (Brandl et al. 2007; Egan et al. 2021). This shows that the *M. subhyalinus* (SE7) specimen we sequenced is part of the *M. subhyalinus* clade more closely related to *M. jeanelli* and *M. falciger* while the previously published *M. subhyalinus* genome is from the clade more closely related to *M. herus,* including our novel *M. herus* (WE3) mitochondrial genome.

To briefly describe the methods, we downloaded all *Macrotermes* COX1 sequences available on GenBank. We also downloaded COX 1 sequences for *Odontotermes longignathus* to serve as an outgroup. Sequences were aligned using MAFFT v7.490 (Katoh and Standley 2013). We partitioned COX1 barcode sequence alignments by codon position. ModelFinder was used to find the best fitting substitution model for each partition (Kalyaanamoorthy et al. 2017). Finally, we used IQ-TREE 2 for maximum likelihood tree inference and assessed support by performing 1000 ultrafast bootstrap replicates (Minh et al. 2020).


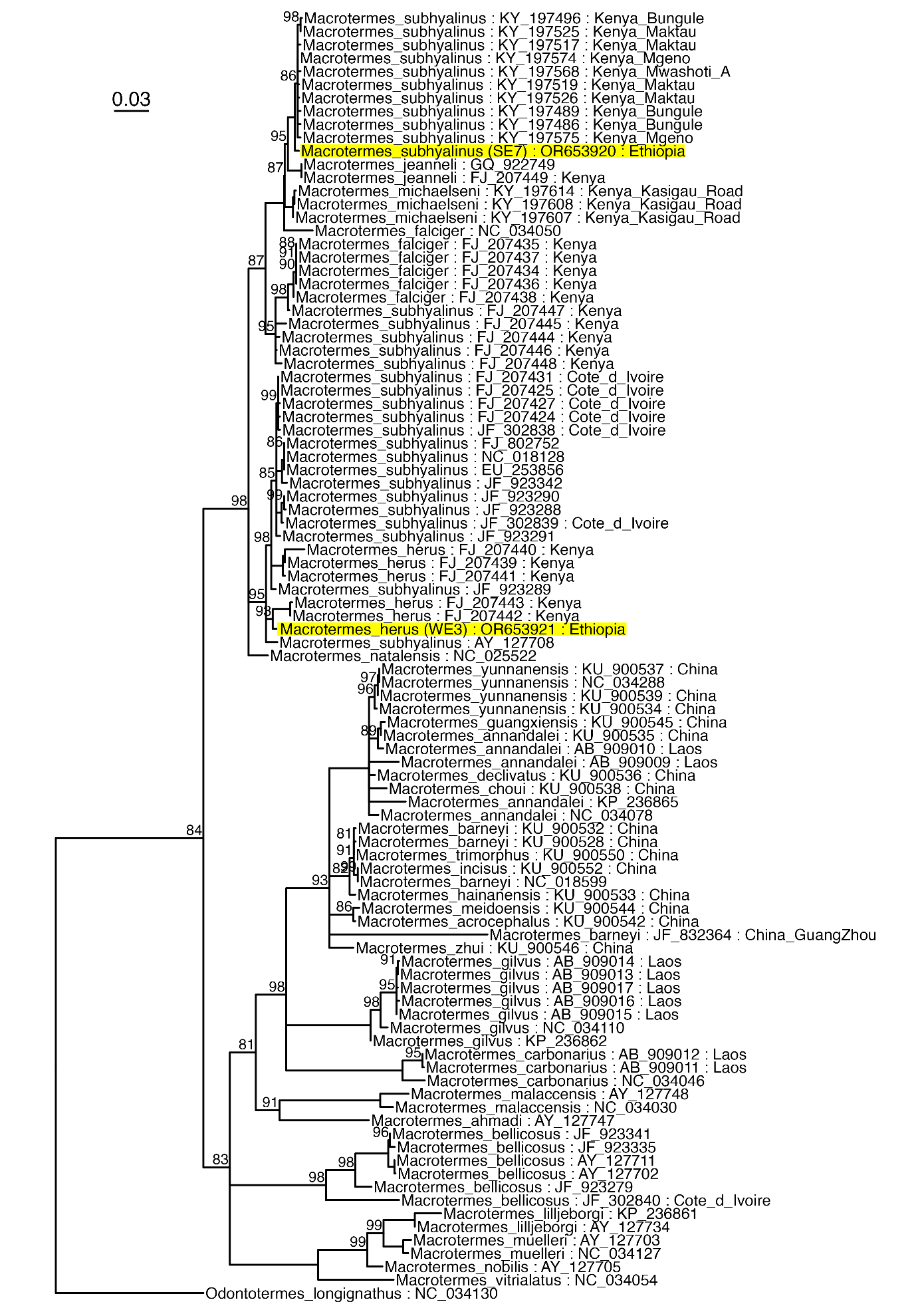


Figure S4: Phylogram showing the maximum likelihood phylogeny of *Macrotermes* based on COX1 barcode sequences. This phylogeny was inferred using IQTREE 2. Ultrafast bootstrap support values are shown on branches. Branches with less than 80% bootstrap support were collapsed, and bootstrap values equal to 100 are omitted for visual clarity. Sequences from Genbank are named using the species name, accession number, and location (when available) separated by colons (:). COX1 sequences from our new mitochondrial genomes are shown at tips ending in solid diamonds (◊). Clades that belong to the same species and that appear monophyletic have been collapsed when distantly related to our new sequences. The black line to the right of the tips of the tree visualizes putative species suggested by the mPTP species delimitation method.
